# Biotechnological applications of *S*-adenosyl-methionine-dependent methyltransferases for natural products biosynthesis and diversification

**DOI:** 10.1186/s40643-021-00425-y

**Published:** 2021-08-11

**Authors:** Congqiang Zhang, Stella Amelia Sultan, Rehka T, Xixian Chen

**Affiliations:** Singapore Institute of Food and Biotechnology Innovation (SIFBI), Agency for Science, Technology and Research (A*STAR), 61 Biopolis Drive, Singapore, 138673 Singapore

**Keywords:** SAM-dependent methyltransferase, SAM-dependent cyclase, Promiscuous methyltransferase, SAM co-factor recycle, SAH inhibition, Methyltransferase assay

## Abstract

In the biosynthesis of natural products, methylation is a common and essential transformation to alter molecules’ bioavailability and bioactivity. The main methylation reaction is performed by *S*-adenosylmethionine (SAM)-dependent methyltransferases (MTs). With advancements in genomic and chemical profiling technologies, novel MTs have been discovered to accept complex substrates and synthesize industrially valuable natural products. However, to achieve a high yield of small molecules in microbial hosts, many methyltransferase activities have been reported to be insufficient. Moreover, inadequate co-factor supplies and feedback inhibition of the by-product, *S*-adenosylhomocysteine (SAH), further limit MTs’ activities. Here, we review recent advances in SAM-dependent MTs to produce and diversify natural products. First, we surveyed recently identified novel methyltransferases in natural product biosynthesis. Second, we summarized enzyme engineering strategies to improve methyltransferase activity, with a particular focus on high-throughput assay design and application. Finally, we reviewed innovations in co-factor regeneration and diversification, both in vitro and in vivo. Noteworthily, many MTs are able to accept multiple structurally similar substrates. Such promiscuous methyltransferases are versatile and can be tailored to design de novo pathways to produce molecules whose biosynthetic pathway is unknown or non-existent in nature, thus broadening the scope of biosynthesized functional molecules.

## Introduction

Methylation plays multiple critical roles, such as diversifying natural products, increasing bioavailability and stability of small molecules, altering the potency and cytotoxicity of natural products, and regulating biological processes, such as epigenetics and initiation of metamorphosis in insects (Shinoda and Itoyama 2003; Niwa et al. 2008; Liscombe et al. 2012; Mo et al. 2017; Li et al. 2018). In nature, the major methyl donor is *S*-adenosylmethionine (SAM), the second most used co-factor that is present in all living organisms to modify a variety of biomolecules from small metabolites to biopolymers (Luo et al. 2019). The major enzyme that depends on SAM is methyltransferase (EC 2.1.1.). The positive charge of the sulfonium atom makes a SAM-dependent reaction mainly an S_N_2 type nucleophilic substitution reaction. SAM-dependent methyltransferases donate the methyl group to a variety of electron-rich chemical groups, such as hydroxyl, alkene or amine groups, and have been classified based on the methyl-accepting group: C-, O-, N- or S-methyltransferases (Struck et al. 2012). To date, there are nearly 400 methyltransferase reactions reported in BRENDA. They are mainly divided in DNA/RNA, protein and small molecule methyltransferases. Approximately half of the methyltransferases catalyse on small molecules. Many small molecules are natural products or secondary metabolites (e.g., terpenoids, alkaloids, flavonoids) which can be used as antibiotics (e.g., nocamycin), flavour and fragrance (e.g., vanillin), energy and fuels (e.g., fatty acid methyl ester) etc. (Katz and Baltz 2016; Kunjapur et al. 2016; Mo et al. 2017; Chen et al. 2019b; Yunus et al. 2020). In this review, we mainly focus on natural product methyltransferases (NPMTs). SAM-dependent methyltransferases are mainly divided into five classes based on their structural folds (Kozbial and Mushegian 2005; Sun et al. 2021). The majority of natural product MTs (NPMTs) belong to class I methyltransferases which have a Rossmann-like superfold (Liscombe et al. 2012). Despite the shared structural folds among the class I methyltransferases, their activities and specificities are very divergent from each other; some catalyse cyclization in addition to methylation reactions (Kim et al. 2011, p. 2; Grocholski et al. 2015; Sun et al. 2021). In some cases, a network of methyltransferases will be able to methylate the same substrate but at different sites of methylation (Mo et al. 2017; Li et al. 2018). The specificity is also contrasted by their ability to accept structurally similar molecules. This permissiveness allows enzyme engineers to modify and improve methyltransferases to methylate related products and design alternative pathways. In this review, we will cover recent advances in biotechnological applications of SAM-dependent methyltransferases for natural product biosynthesis and diversification. It generally involves three important aspects: first, to identify the desired methyltransferases in natural product biosynthetic pathway; second, to engineer higher methyltransferase activities through enzyme engineering and high-throughput screening; finally, to improve co-factor regeneration. We will discuss all three aspects in the following sections.

## Main text

### Identifying the desired methyltransferase activity

**Fig. 1:**
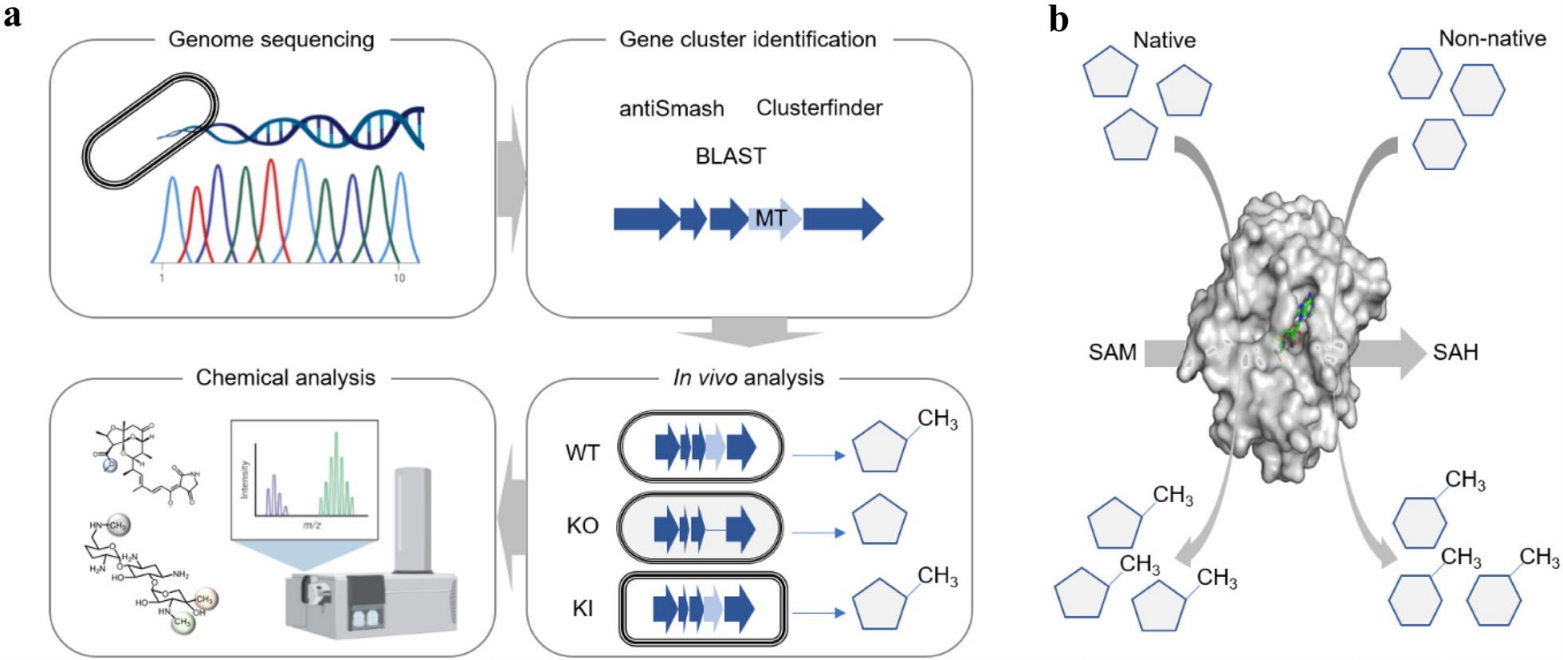
Illustrations of methods to identify the desired methyltransferases activities. **a** Genomic and chemical screening methods are used to identify the novel methyltransferases from native microbial hosts. First, genome sequencing is carried out to obtain the genetic information. Second, in silico tools are used to identify the putative methyltransferase gene. For natural product biosynthesis, such methyltransferases are often found in a biosynthetic gene cluster in microbial hosts. Third, mutant strains with knock-out (KO) or knock-in (KI) to delete or overexpress the putative methyltransferases gene, respectively, are constructed. Finally, chemical screening is then carried out to profile the non-methylated intermediates or elevated methylated products from these mutant strains as compared to wildtype (WT) strain. Some illustrations were created with BioRender.com. **b** Promiscuous methyltransferases are used to catalyze structurally similar substrates to achieve the desired methylation reaction. The 3D model is caffeate O-methyltransferase from *Homo sapiens* (PDB: 3BWY). The bound SAH is shown in green

Biosynthesis of small molecules in heterologous hosts through metabolic engineering and synthetic biology has been significantly developed (Cravens et al. 2019; Chen et al. 2019b; Yang et al. 2020). One important prerequisite is to delineate the biosynthetic pathway and identify the enzymes to produce the desired natural product. Predominantly, gene discovery from the native hosts is commonly used, especially with the advancement in genome sequencing, genome modification and bioinformatics (Katz and Baltz 2016) (Fig. 1a). This method usually requires access to the native hosts, and performing genome sequencing and metabolite profiling (Katz and Baltz 2016). The other method is to employ promiscuous enzymes that catalyse structurally similar substrates to perform the desired biotransformation (Lin et al. 2019; Huffman et al. 2019) (Fig. 1b). It relies on existing knowledge of identified natural enzymes and expands the substrate scope of natural enzymes (Li et al. 2020). In this section, we will cover the recent discovery of novel methyltransferases and the application of promiscuous methyltransferases for natural product biosynthesis (Table 1).

**Table 1:** Summary of recently discovered methyltransferases

Product	Enzyme	Name	Reaction type	Natural host	Gene cluster/biosynthetic pathway	Discovery method	Promiscuity	Refs.
Novel methyltransferases discovery								
Nonamycin I	NcmP	Carboxylate O-methyltransferase	Methylation	*Saccharothrix syringae NRRL B-16468*	Tetramic acid, nocamycin biosynthetic cluster	Biosynthetic gene cluster identification. mutant strain with NcmP disruption by λ-RED recombination	Specific methylation site, methylate the pathway intermediates	Mo et al. (2017)
Antibiotic CC1065	C10P	Raidcal SAM enzyme	Radical generation and methylation	*Shewanella woodyi ATCC 51908*	Spirocyclopropylcyclohexadienone biosynthesis cluster	Benzodipyrrole Biosynthetic gene cluster identification. Gene inactivation and re-introduce the same gene, and intermediate characterization	N.A	Jin et al. (2018)
	C10Q	Methyltransferase	Methylation and cyclization	*S. Zelensis nrrl 11183*			N.A	
Xantholipin	XanM1	O-methyltransferase	Methylation	*Streptomyces flavogriseus*	Aromatic polyketides, Polycyclic xanthones biosynthetic cluster	Biosynthetic gene cluster identification. In vivo gene deletion study	N.A	Kong et al. (2020)
	XanM2	O-methyltransferase	Methylation				Specific methylation site, methylate the pathway intermediates	
	XanM3	O-methyltransferase	Methylation					
Gentamicin C	GenN	N-methyltransferase	Methylation	*Micromonospora echinospora*	Aminoglycosides, Gentamicin C biosynthesis cluster	Biosynthetic cluster identification. Systematic deletion of the methyltransferase genes. knockout the biosynthetic gene cluster and complement with plasmids containing the MT genes one by one and perform in vivo biotransformation	Specific methylation site, methylate the pathway intermediates	Li et al. (2018)
	GenD1	C-methyltransferase	Methylation					
	GenK	C-methyltransferase	Methylation					
	GenL	Terminal 6′-N-methyltransferase	Methylation		Outside the gentamicin C biosynthetic cluster	Genome sequencing. BLAST search. Enzyme screening to identify genL. Gene deletion to confirm in vivo activity. In vitro enzyme characterization		
Pre-sodorifen pyrophosphate	SodC	C-methyltransferase	Methylation and cyclization	*Serratia plymuthica WS3236*	Sodorifen biosynthetic gene cluster	Genome sequencing. In silico methods (antiSMASH and ClusterFinder) to identify the biosynthetic gene cluster. Overexpress the gene cluster in *E. coli*	N.A	Duell et al. (2019)
C6,C7 prenyl pyrophosphate	IPPMT	Isoprenyl pyrophosphate methyltransferase	Methylation	*Streptomyces monomycini*	Terpenoids biosynthetic cluster	BLAST GPPMT with genome sequences of *S. monomycini.* Overexpression and purification from *E. coli* for activity characterization	Multiple methylation	Drummond et al. (2019)
Teleocidin B	TleD	C-methyltransferase	Methylation and cyclization	*Streptomyces mediocidicus*	Indole alkaloid, Teleocidin B biosynthesis cluster	BLAST search to identify teleocidin B biosynthesis cluster. overexpress the gene cluster in *Streptomyces lividans TK21*	Produce a mixture of isomers	Awakawa et al. (2014)
Promiscuous methyltransferase applications								
Vanillin	COMT	Caffeic acid O-methyltransferase	Methylation	*Homo sapiens*	*Escherichia coli*	Natural substrate is caffeic acid		Kunjapur et al. (2016)
Pterostilbene	COMT			*Arabidopsis thaliana*				Heo et al. (2017)
Fatty acid methyl esters	DmJHAMT	Juvenile hormone acid O-methyltransferase	Methylation	*Drosophila melanogaster*	Juvenile hormone biosynthesis	Homolog, sequence alignment	Broad specificity for medium chain free fatty acids	Sherkhanov et al. (2016)

#### Discovery of novel methyltransferases from native hosts

**Fig. 2:**
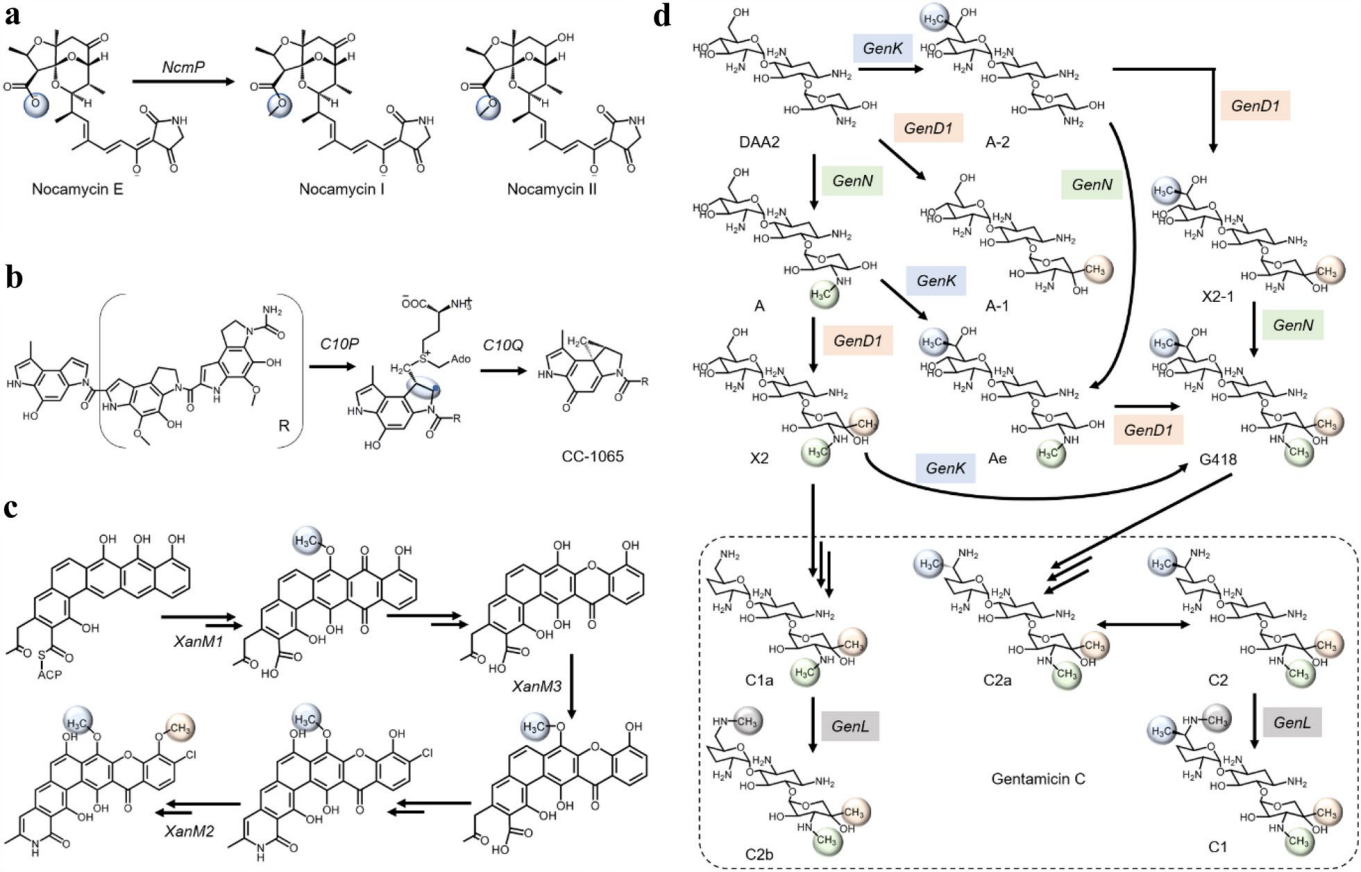
Reactions of recently discovered novel methyltransferases. These enzymes are mainly discovered through genomic and chemical screening (Table 1). **a** Methylation of nocamycin E by NcmP to produce nocamycin I. The structure of Nocamycin II is shown. **b** Novel radical-SAM mediated cyclopropanase system to produce the antibiotic CC1065. **c** Network of methyltransferases involved in xantholipin biosynthesis. **d** Network of methyltransferases involved in gentamicin C biosynthesis. The starting compound is 3-dehydro-3-oxo-gentamicin A2 (DAA2)

Based on our survey of recent literature, the majority of novel methyltransferases characterized have been discovered from microbes and are located in biosynthetic gene clusters (Chavali and Rhee 2017; Soldatou et al. 2019) (Table 1). Thus, we will mainly focus on novel methyltransferases discovered from microorganisms. To elucidate the functions of methyltransferase genes, genomic and chemical screening are commonly used. In general, the methyltransferase gene is disrupted in the native microbial host, and the metabolite profiles between the wild-type and mutant strain will be compared to identify the accumulated intermediates via accurate mass analysis (Fig. 1a). Using this strategy, Mo et al. (2017)have identified the carboxylate O-methyltransferase (NcmP) from *Saccharothrix syringae NRRL B-16468*, which methylates nocamycin E to produce nocamycin I, a potent antibiotic molecule (Fig. 2a). The authors hypothesized that nocamycin II, which is structurally similar to nocamycin I, was also formed by the action of NcmP. In another study, genomic and chemical profiling have elucidated a novel two-component cyclopropanase system to synthesize antibiotic CC1065 from *Shewanella woodyi ATCC 51908* (Jin et al. 2018). The cyclopropanase system comprises C10P, a radical-SAM enzyme and C10Q, a bifunctional methylase and cyclase (Wu et al. 2017; Jin et al. 2018). By deleting either *C10P* or *C10Q*, the mutant strain failed to produce the antibiotic CC1065. Labelling experiments suggested that C10P generated SAM methylene radical and formed SAM-substrate covalent adduct which was biotransformed by C10Q to cyclopropane moiety (Fig. 2b). Cyclopropane moiety is challenging for synthetic chemists and often presents in clinical drugs (Jin et al. 2018). Discovering or engineering cyclopropane-forming enzymes may thus provide alternative synthesis routes for pharmaceutical applications. In another study, Kong and co-authors elucidated the functions of three methyltransferases (XanM1-3) in the polyketide compound, xanthones, biosynthetic cluster from *Streptomyces flavogriseus* (Fig. 2c). However, when XanM1 was deleted, neither the product nor intermediate was detected, leading the authors to hypothesize that XanM1 catalysed an intermediate that was possibly still tethered to the acyl carrier protein of polyketide complex. Interestingly, purified XanM1 is able to methylate the substrates of XanM2 and XanM3, and the three methyltransferases share minor overlapping methylation activities towards several intermediates along the xantholipin pathway (Kong et al. 2020). This led the authors to identify a common ancestor for the three methyltransferases and postulated that *XanM1-3* have been evolved through gene duplication. As shown in Fig. 2, many of the natural products and intermediates have complex chemical structures, thus they may not be readily available commercially. Isolating these compounds from mutant strains is necessary to confirm the function of the methyltransferase enzyme. In a recent work by Li et al. (2018), the intermediates along the gentamicin C biosynthetic pathway were purified from *Micromonospora echinospora* mutant strains to probe the function of a complex methyltransferase network (Fig. 2d)*.* In addition to metabolite profiling of methyltransferase-deleted strains, the author overexpressed individual methyltransferase (*genN*, *genD1* and *genK*) in *M. echinospora* with gentamicin biosynthetic cluster deleted. The isolated pathway intermediates were then added separately into the culture media of the strain overexpressing each of the methyltransferase genes to identify the methylated product. With this approach, Li and colleagues provided strong evidence that these methyltransferases display strong selectivity towards the site of methylation but readily accept several analogous pathway intermediates. Unexpectedly, the authors have also discovered an N-methyltransferase (*GenL*) that catalyses the essential last-step 6′-N-methylation, which is not clustered together with the other three MTs on the genome (Fig. 2d). Even though GenL is active to convert gentamicin C1a and C2 to C2b and C1, respectively, the question of the primary function of GenL remains open.

**Fig. 3:**
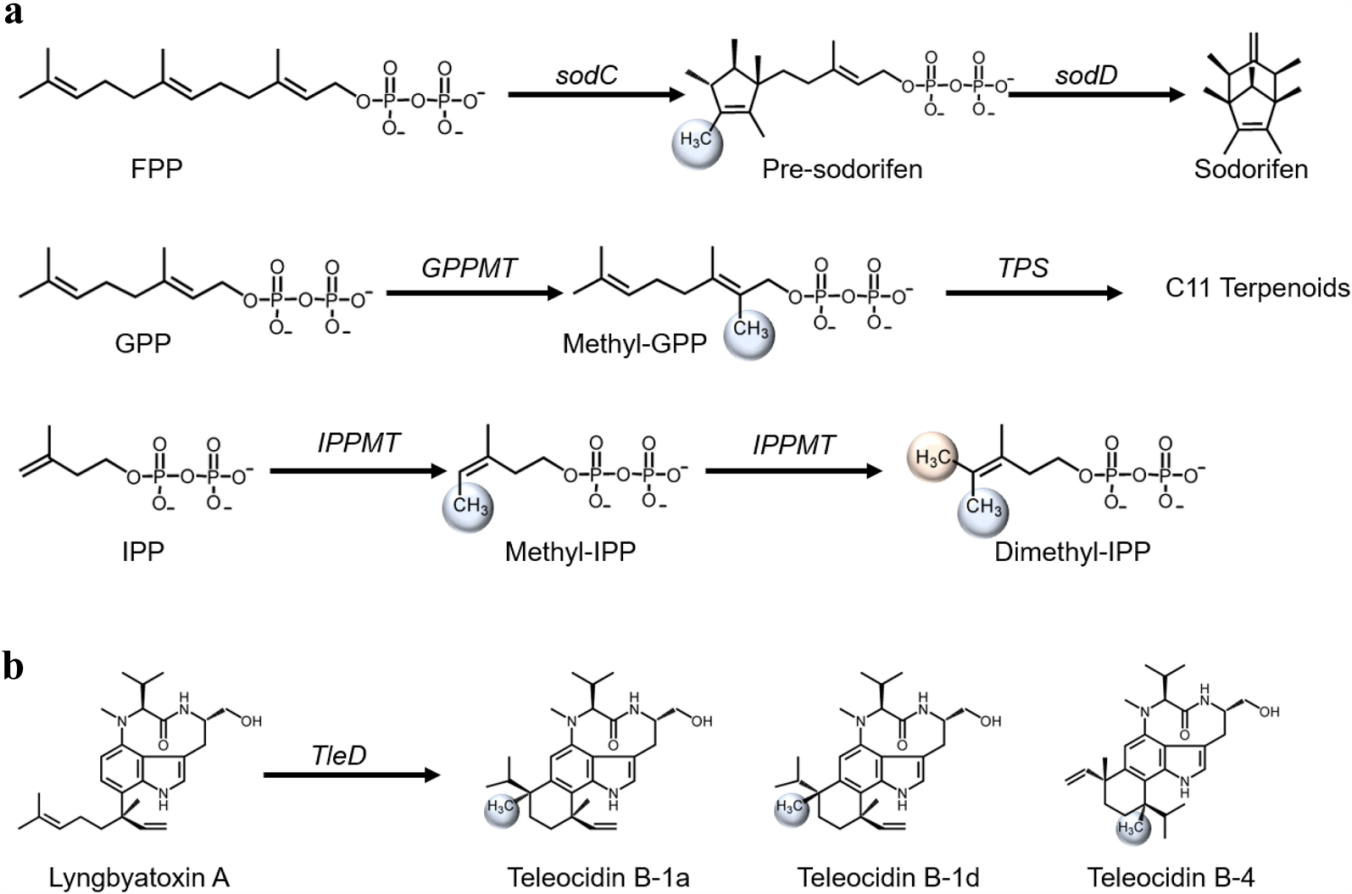
Reactions of recently discovered novel methyltransferases. In silico tools have been used to discover these enzymes (Table 1). **a** Methyltransferase reactions that modify terpenoid precursors. IPP, isopentenyl pyrophosphate; GPP, geranyl pyrophosphate; FPP, farnesyl pyrophosphate. **b** Bifunctional methyltransferase and cyclase involved in teleocidin biosynthesis

All the previous examples of methyltransferases discovery started with genome mining and biosynthetic gene cluster identification (Table 1). Predicting the biosynthetic gene cluster of natural products has been greatly facilitated by the advancement in in silico prediction tools (Medema et al. 2011; Chavali and Rhee 2017; Soldatou et al. 2019). Duell et al. (2019) applied antiSMASH and ClusterFinder to find the sodorifen biosynthetic gene clusters in *Serratia plymuthica WS3236* (Medema et al. 2011; Cimermancic et al. 2014)*.* Sodorifen is synthesized from farnesyl pyrophosphate (FPP) by a bifunctional methyltransferase and cyclase (SodC) and a terpene cyclase (SodD) (Reuss et al. 2018; Duell et al. 2019) (Fig. 3a). The enzymatic activities were verified by overexpressing *SodC* and *SodD* genes in *Escherichia coli* with an elevated amount of FPP, and directly analysing the products formed (Duell et al. 2019). In another study, Drummond et al. (2019) used geranyl pyrophosphate (GPP) methyltransferase from *Streptomyces coelicolor* as a query to BLAST search for natural variants in bacteria and identified an IPP methyltransferase (IPPMT) from *Streptomyces monomycini* (Fig. 3a). Noteworthy, the native host does not produce any methylated terpenoid compounds. In such case, the previous method of gene knockout and intermediate identification will not be able to verify the function of the gene. To circumvent this, the biosynthetic gene cluster can be overexpressed in a related species with low or no secondary metabolite production, and screen for the products formed with the additional genes (Ahmed et al. 2020) (Fig. 1a). For example, Awakawa et al. (2014) overexpressed the indole alkaloid, teleocidin B, biosynthetic pathway, from *Streptomyces mediocidicus,* in *Streptomyces lividans TK21* which does not produce teleocidin B*.* This led the authors to discover a bifunctional methyltransferase and cyclase, TleD, that could methylate and cyclize the terpene moiety of teleocidin B (Yu et al. 2016) (Fig. 3b).

Such discovery of novel methyltransferases which modify complex secondary metabolites continues to expand our biocatalysts toolbox to biosynthesize more diverse natural products. Most of the methyltransferases possess promiscuous activities, which potentially can be applied to accept new substrates, broadening the biocatalytic diversity.

#### Applications of promiscuous methyltransferases

**Fig. 4:**
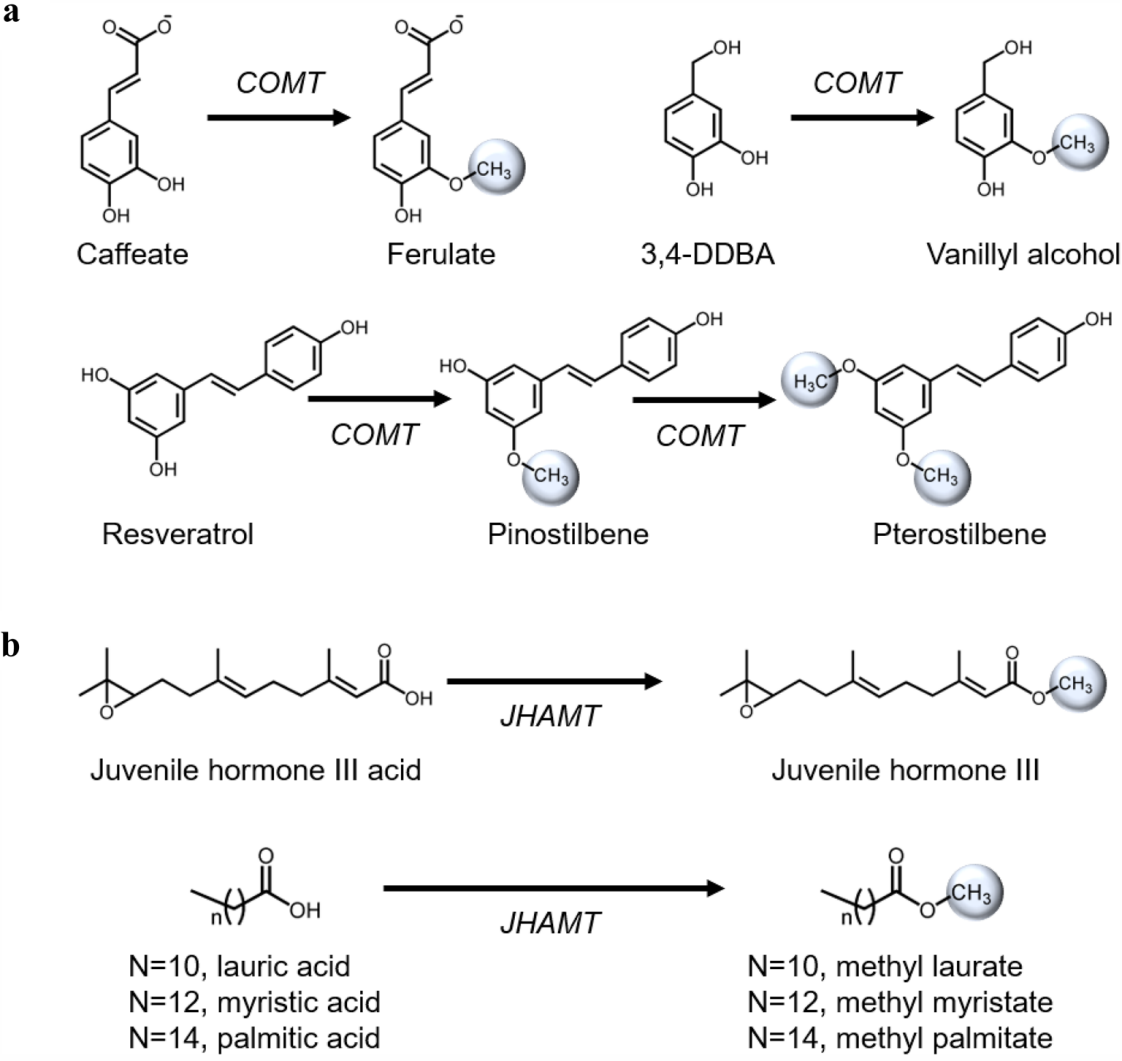
Reactions of promiscuous methyltransferases for small molecule production. **a** Caffeate O-methyltransferases (COMT) readily accept structurally similar compounds, such as caffeate, 3,4-dihydroxybenzyl alcohol (3,4-DDBA) and resveratrol. **b** Juvenile hormone acid O-methyltransferase (JHAMT) can methylate C12–C16 fatty acid. Refer to Table 1 for more information on the enzymes

Promiscuous methyltransferases can be applied to accept similar but non-native substrates to produce desired methylated products (Fig. 1b). De novo pathway could be designed with promiscuous enzymes to biosynthesize compounds whose pathways are unknown (Lin et al. 2019; Li et al. 2020). One such important promiscuous methyltransferase is caffeic acid O-methyltransferase (COMT). Promiscuous COMT from *Homo sapiens* (Hs.COMT) was able to perform a key 4-O-methylation reaction in the vanillin biosynthesis pathway (Kunjapur et al. 2016; Chen et al. 2017). Chen et al. (2017) has demonstrated that Hs.COMT methylate the alternative substrate 3,4-dihydroxybenzyl alcohol (3,4-DDBA) at a reasonable efficiency and produce ~ 500 mg/L vanillyl alcohol by whole-cell biotransformation (Fig. 4a). 3,4-DDBA is a smaller substrate as compared to caffeic acid, and hence, it is conceivable that the binding pocket of Hs.COMT can accept 3,4-DDBA, although the binding affinity is not strong (*K*_m_ = 0.52 mM) (Chen et al. 2017). Interestingly, COMT’s active site can accommodate bigger substrates. Heo et al. (2017) have identified such COMT from *Arabidopsis thaliana* (At.COMT) to bind resveratrol (*K*_m_ = 44.9 µM) with equivalent affinity as caffeic acid (*K*_m_ = 40.5 µM) (Fig. 4a). With At.COMT, the authors established a de novo pathway to produce ~ 33 mg/L di-methylated resveratrol, pterostilbene, in *Escherichia coli *(*E. coli*). The mono-methylated resveratrol, pinostilbene, was accumulated, indicating further optimization of At.COMT activity is required.

Fatty acid methyl ester (FAME) is a renewable, biodegradable, and environmentally friendly biofuel. However, toxic methanol is used to chemically convert fatty acid to FAME. Yunus et al. (2020) employed a juvenile hormone acid O-methyltransferase from *Drosophila melanogaster* (DmJHAMT) and developed a methanol-free biosynthesis route to produce FAME (Fig. 4b). DmJHAMT is a key regulatory enzyme for insect metamorphosis and displays a broad substrate spectrum ranging from C12 to C16 fatty acids (Shinoda and Itoyama 2003; Sherkhanov et al. 2016). By fusing DmJHAMT downstream of a strong promoter and increasing intracellular SAM concentration, > 95% lauric acid was converted to methyl laurate (Yunus et al. 2020). This biotransformation strategy is promising to replace the toxic chemical process. Even though high conversion yield was achieved, the DmJHAMT is still one of the main limiting factors that requires further optimization.

Engineering of promiscuous enzymes is required to increase their specificity and activity towards desired substrates, and enzyme engineering strategies such as structural guided mutagenesis are often used (Chen et al. 2019a; Chen and Arnold 2020; Li et al. 2020). Moreover, high-throughput methyltransferase assays will significantly improve the rate of identifying the beneficial mutants, which will be discussed in the next section.

### Engineering methyltransferase activity

To identify beneficial mutations from the astronomical size of protein sequences, computer-aided structural analysis can provide insights and guide rational mutant designs. For example, to improve the activity of bergaptol O-methyltransferase (BMT), Zhao et al. determined the crystal structure of BMT from *Peucedanum praeruptorum* (Pp.BMT) and rationally designed 14 single mutants by selecting low mutation energies calculated by Discovery Studio 4.1 (Zhao et al. 2020). One of the mutants, V320I, improved the enzymatic activity by > eightfold. When a crystal structure is not available, homology models are constructed based on sequence similarity. Ignea and colleagues constructed a homology model for GPP methyltransferase from *Pseudanabaena limnetica* (Pl.GPPMT) and designed 44 mutants (Ignea et al. 2018). Among them, two single substitutions, V250A and F226H, improved C11 terpenoid production by two- and threefold, respectively (Fig. 3a). Although computer-aided enzyme design has advanced significantly, it is still a resource intensive process to screen just a fraction (10^5^–10^6^) of protein sequence space computationally (Wu et al. 2019). Often, in silico method is combined with robust and sensitive high-throughput screening (HTS) assay to speed up the engineering process. In this section, we will summarize HTS assays developed for small molecule methyltransferases (Table 2). DNA or protein methyltransferase assays have been reviewed in previous reports, although some assays can be applied for NPMT screening too (Luo 2012; Li et al. 2017; Zhang et al. 2021).

**Table 2:** Summary of high-throughput assays for methyltransferase

Target MT	Detection molecule	Modality class	Enzyme or reagent/reporter	Measurement	Detection limit	Dynamic range	Throughput	Remarks	Refs.
Salicylic acid carboxyl methyltransferase	SAH → Homocysteine	Coupled-enzyme reaction	SAH nucleosidase and *S*-ribosylhomocysteine lyase/5,5′-dithiobis-2-nitrobenzoic acid (DTNB, Ellman's reagent)	Absorbance	20 µm SAH		Multi-well plate format	For purified MTs	Hendricks et al. (2004)
Putrescine N-methyltransferase	SAH → Homocysteine	Coupled-enzyme reaction	SAH nucleosidase and S-ribosylhomocysteine lyase/DTNB, Ellman’s reagent	Absorbance	20 µm SAH	0–115 µm SAH (*R*^2^ = 0.9963)	Multi-well plate format	SAM degradation leads to TNB production For purified MTs	Biastoff et al. (2006)
Protein arginine N-methyltransferase	SAH → hypoxanthin	Coupled-enzyme reaction	SAH nucleosidase, adenine deaminase	Absorbance	10 µm substrate	0–100 µm SAH	Multi-well plate format	Interference from protein which has absorbance at 280 nm For purified MTs	Dorgan et al. (2006)
Histone methyltransferase and M.haeiii	SAH → ammonia	Coupled-enzyme reaction	SAH nucleosidase, adenine deaminase and glutamate dehydrogenase/NADPH	Absorbance	170 nm SAH	0–7000 µm peptide concentration	Multi-well plate format	Ammonia contamination from reaction needs to be avoided For purified MTs	Duchin et al. (2015)
Catechol-O-methyltransferase	SAH → homocysteine	Coupled-enzyme reaction	SAH hydrolase/fluorescein–cystamine–methyl red (FL-S–S-MR)	Fluorescence	≤ 1 µm homocysteine	0–50 µm homocysteine (*R*^2^ = 0.995)	Multi-well plate format	For purified MTs	Wang et al. (2005)
Salicylic acid methyltransferase	SAH → H_2_O_2_	Coupled-enzyme reaction	SAH nucleosidase, xanthine oxidase, and horseradish peroxidase enzymes/Amplex Red (10-acetyl-3,7-dihydroxyphenoxazine)	Fluorescence	36 nm for salicylic acid	0–10 µm salicylic acid (*R*^2^ = 0.94)	Multi-well plate format	For purified MTs	Akhtar et al. (2018)
Protein arginine methyltransferases, histone-lysine N-methyltransferase and a sarcosine/dimethylglycine N-methyltransfease	SAH → SIH	Coupled-enzyme reaction	Deaminase TM0936	Absorbance	2.35 µm substrate (absorbance)	2.35–46.96 µm SAM	Multi-well plate format	interference from protein which has absorbance at 280 nm For purified MTs	Burgos et al. (2017)
Protein arginine methyltransferases, histone-lysine N-methyltransferase and a sarcosine/dimethylglycine N-methyltransfease	SAH → SIH	Coupled-enzyme reaction	Deaminase TM0936/*S*-8-aza-adenosyl-l-methionine (8-aza-SAM)	Fluorescence	25 µm substrate	Three logs of linear dynamic range	Multi-well plate format	8-aza-SAM is a good substrate for most MT For purified MTs	
Histone methyltransferase	SAH	RNA aptamer/riboswitch	3,5-difluoro-4-hydroxybenzylidene imidazolinone (DFHBI)	Fluorescence	75 nm SAH tested	Tens of nanomolar to tens of micromolar	Flow cytometry	Bind to ATP abd NAD + in vitro, although it has low fluorescence level In vivo assay	Su et al. (2016)
Phenylethanolamine N-methyltransrease (PNMT) Acetylserotonine O-methyltransferase [Asmt]	SAH → cysteine	Coupled-microbial growth Adaptive laboratory evolution	Delete serine acetyltransferase (cysE), overexpress cystathionine-β-synthase (cys4) and cystathionine-γ-lyase (cys3)	Absorbance			10 million cells per passage	In vivo assay False positive rate may be high, due to improvement in native MTs activity	Luo et al. (2019)
Catechol-O-methyltransferase	Vanillate	Transcription factor	Caulobacter crescentus VanR-VanO	Fluorescence	0.01 mm Vanillate	0.01–1 mm vanillate	Flow cytometry	In vivo assay Only specific to vanillate synthesis	Kunjapur and Prather (2019)
Commercial kit	SAH	RNA aptamer/riboswitch	Tb-Streptavidin and dylight650	Time-resolved fluorescence resonance energy transfer	0.6 nm SAH	0.6–2500 nm SAH	Multi-well plate format	For purified MTs	
Commercial kit	SAH → ATP	Coupled-enzyme reaction	MTase-Glo reagent and MTase-Glo detection reagent	Luminescence	50 nm SAH	0–10 µm SAH (*R*^2^ > 0.99)	Multi-well plate format	For purified MTs	
Commercial kit	SAH → H_2_O_2_	Coupled-enzyme reaction	SAH nucleosidase, adenine deaminase, xanthine oxidase, and horseradish peroxidase enzymes/amplex Red (10-acetyl-3,7-dihydroxyphenoxazine)	Fluorescence	1.25 µm Resorufin	0–10 µm Resorufin	Multi-well plate format	For purified MTs	
Commercial kit	SAH	Antibody	SAH-d2 and Lumi4-tb cryptate conjugated antibody	Time-resolved fluorescence resonance energy transfer	30 nm SAH	10–1000 nm SAH	Multi-well plate format	For purified MTs	Kimos et al. (2016)

#### In vitro high-throughput assay

Most methyltransferase assays are designed to quantify the by-product, SAH. Coupled-enzyme reaction is frequently applied to convert SAH into chromogenic or fluorescent molecules (Table 2). The advantage of such design is that it removes any SAH inhibition to methyltransferases. Usually, the enzymes in the methionine cycle are utilized. For example, SAH nucleosidase (mtn) and *S*-ribosylhomocysteine lyase (LuxS) readily convert SAH to homocysteine, which contains a free thiol group. Ellman’s reagent (5,5′-dithiobis-2-nitrobenzoic acid) has been applied to quantify the concentration of thiol groups, and hence, it is used to quantify homocysteine concentration. The resulting yellow solution has an absorbance at 412 nm, which increases linearly with increasing SAH concentration (Hendricks et al. 2004; Biastoff et al. 2006). Similarly, a thiol-activated fluorescent reporter molecule, fluorescein–cystamine–methyl red (FL–S–S–MR), has been synthesized to quantify homocysteine concentration (Wang et al. 2005). However, it is to note that these assays are sensitive to the presence of reducing agents such as dithiothreitol (DTT) or cysteine residues present in enzymes, leading to high background readings. These assays are thus mainly applied to characterize purified methyltransferases. Another moiety of SAH is the nucleobase adenine. When adenine is deaminated by adenine deaminase to hypoxanthin, a decrease in UV absorbance at 265 nm is observed. The change in absorbance at 265 nm is visible at as low as 10 µM adenine (Dorgan et al. 2006). In fact, when SAH is deaminated by deaminase TM0936 to form *S*-inosylhomocysteine (SIH), a discernible drop in absorbance at 263 nm is observed, reducing the number of additional enzymes required to convert SAH into chromogenic substrate (Burgos et al. 2017). The decrease in 263 nm absorbance can be monitored continuously. The assay has been applied to detect and characterize glycine N-methyltransferase activity in rat liver extracts, and the lowest activity detected was 2 μM/h. To improve the assay sensitivity and avoid the inference from biomolecules whose absorbance is around 260–280 nm, the study developed another fluorescence-based assay using a SAM analogue, 8-aza-SAM. However, the availability of this analogue may prohibit its usage (Burgos et al. 2017). Moreover, adenine can be converted to dihydroxyadenine by xanthin oxidase (XOD). Hydrogene peroxide (H_2_O_2_) is generated during the reaction and subsequently utilized by horseradish peroxidase to oxidize amplex red to a fluorescent molecule, resorufin (Akhtar et al. 2018). The assay has been commercialized (Cayman Chemical #700150), although the XOD activity is insufficient and results in slow channelling of SAH to dihydroxyadenine (Burgos et al. 2017). All the assays have been developed to characterize purified methyltransferases. The sensitivity might be significantly reduced when applying the assays to semi-purified methyltransferases because of the presence of interfering substances. To our knowledge, none of the assays have been applied to screen for methyltransferase variants, since the throughput may be compromised if enzyme purification is required.

#### In vivo high-throughput assay

**Fig. 5:**
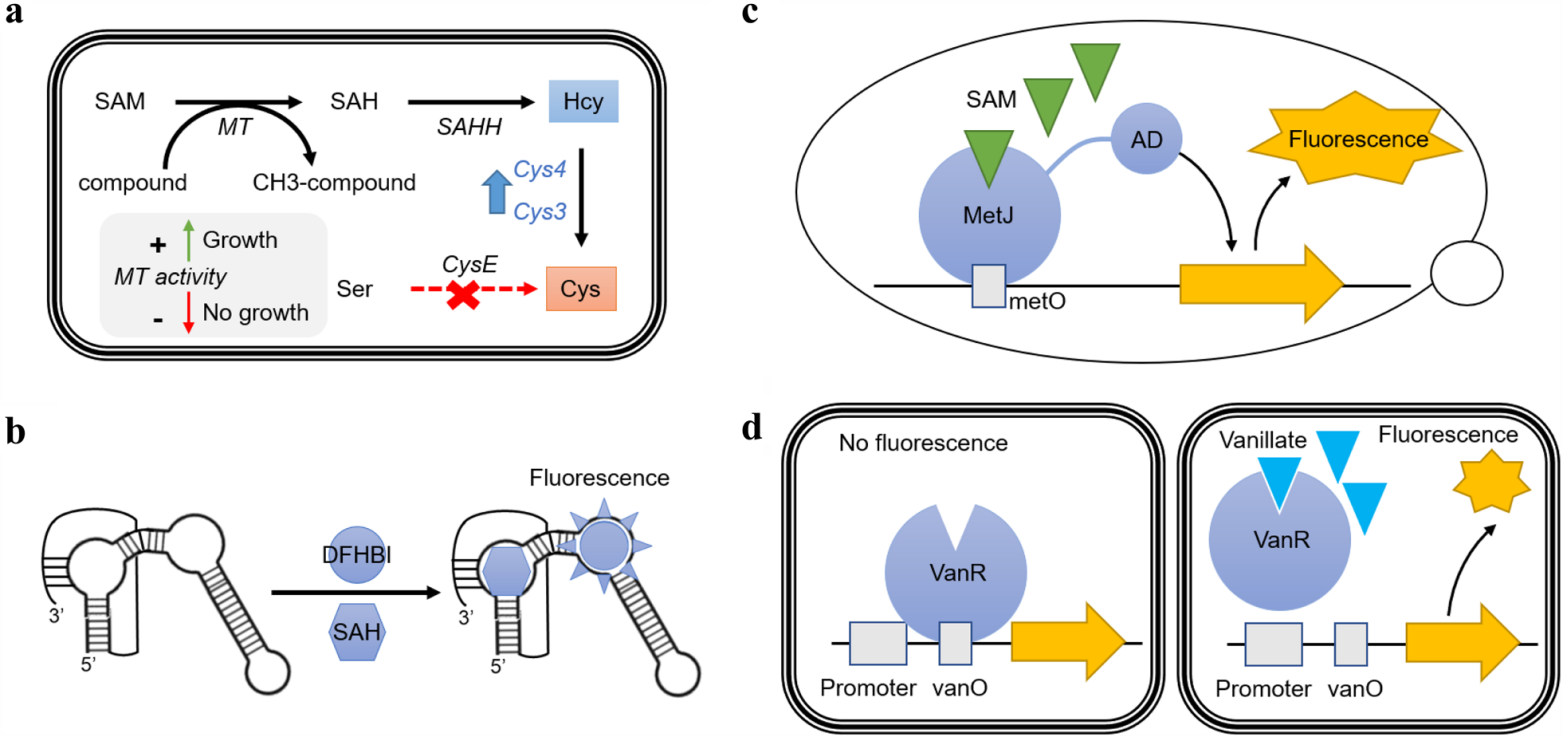
Illustrations of in vivo high-throughput methyltransferases assays. **a** Growth-coupled methyltransferase assay, where the essential amino acid cysteine (Cys) biosynthesis is linked to SAH biosynthesis. SAH concentration is dependent on methyltransferase activity. If methyltransferase activity is present, Cys will be produced and there is rapid microbial growth. If methyltransferase activity is absent, Cys will not be produced, and no microbial growth is observed. **b** SAH riboswitch-based biosensor which will bind to 3,5-difluoro-4-hydroxybenzylidene imidazolinone (DFHBI) in the presence of SAH and produce fluorescence signal. **c** MetJ-MetO system as a biosensor to quantify intracellular SAM concentration in *S. cerevisiae.* MetJ is fused with an activating domain (AD). In the presence of SAM, metJ will bind to metO and AD will activate the downstream reporter protein expression. **d** VanR-VanO system as a biosensor to quantify vanillate concentration. In the absence of vanillate, VanR binds to VanO and blocks transcription. In the presence of vanillate, VanR will not be able to bind to VanO, and downstream reporter gene transcription will proceed. Please refer to Table 2 for more information

To increase the throughput, clarified cell lysate or in vivo assays are preferred. Luo et al. (2019) have designed a growth-coupled in vivo methyltransferase assay by linking essential cysteine biosynthesis to the methylation byproduct SAH (Fig. 5a). The assay can screen 10^7^ mutants at one time, significantly increasing the throughput. With the growth-coupled assay, the authors successfully identified a single mutation (F214L) in phenylethanolamine N-methyltransrease (PNMT) which improved the PNMT’s activity against a non-natural substrate octopamine by twofold. Moreover, the study showed that the in vivo assay can be applied to optimize a heterologous pathway in *E. coli* involving methylation. One drawback of the assay is the potentially high false positives, as the study identified that SAM-dependent cfa from *E. coli*, instead of PNMT, was improved in non-growth-coupled isolates. Moreover, growth rate enhancement is a complex and non-specific trait that may not have high dynamic range (Lin et al. 2017). Recently, biosensors have advanced to enable high-throughput measurement of target molecule in vivo. Natural SAH riboswitches have been reported which can distinguish SAH from SAM by > 1000-fold (Wang et al. 2008). Su et al. (2016) screened 58 RNA riboswitches for SAH and identified two promising biosensors (Nmo1-4 from *Nitrococcus mobilis*, Mpe1-5 from *Methylibium petroleiphilum*) to quantify SAH concentration both in vitro and in vivo (Fig. 5b). The biosensors have a dynamic range from nanomolar to micromolar concentration of SAH, which nicely captures intracellular SAH concentration (~ 1.3 µM). The study showed that when SAH nucleosidase (mtn) activity was inhibited in *E. coli* BL21, the biosensor Nmo1-4 gave rise to fluorescence signals that were directly proportional to the concentration of mtn inhibitors, implying that the biosensor could estimate SAH concentration in vivo. In addition to RNA aptamer, proteins or transcription factors that respond to small molecules are also used as biosensors to quantify methyltransferase activity. MetJ-MetO system, which is described in the subsequent section, has been developed in *S. cerevisiae* to sense intracellular SAM concentration (Umeyama et al. 2013; Dong et al. 2021) (Fig. 5c). Another example is the vanillate biosensor that has been optimized to assay O-methyltransferase activity for vanillate production (Fig. 5d). It is based on a natural *Caulobacter crescentus* VanR-VanO system and achieved 14-fold dynamic range (Meyer et al. 2019; Kunjapur and Prather 2019). With the biosensor, the author screened 16 natural O-methyltransferase variants from bacterial, fungal and archaeal sources and identified three novel O-methyltransferases that are more active than commonly used Hs.COMT. These biosensors are promising biotechnological tools to be applied to evolve methyltransferases in a high-throughput manner, which awaits to be demonstrated, although one important pre-requisite is that the methyltransferase substrate must be uptaken into the cell. Unfortunately, product-specific biosensors lack universal application to probe other NPMTs. In addition, optimizing the small-molecule-responsive biosensor requires significant engineering efforts. In comparison, detecting the by-product SAH might be more applicable to measure most methyltransferase activities, although it will fail to quantify non-methylating methyltransferase activities (Fage et al. 2015; Ohashi et al. 2017).

### Co-factor regeneration and diversification

**Fig. 6:**
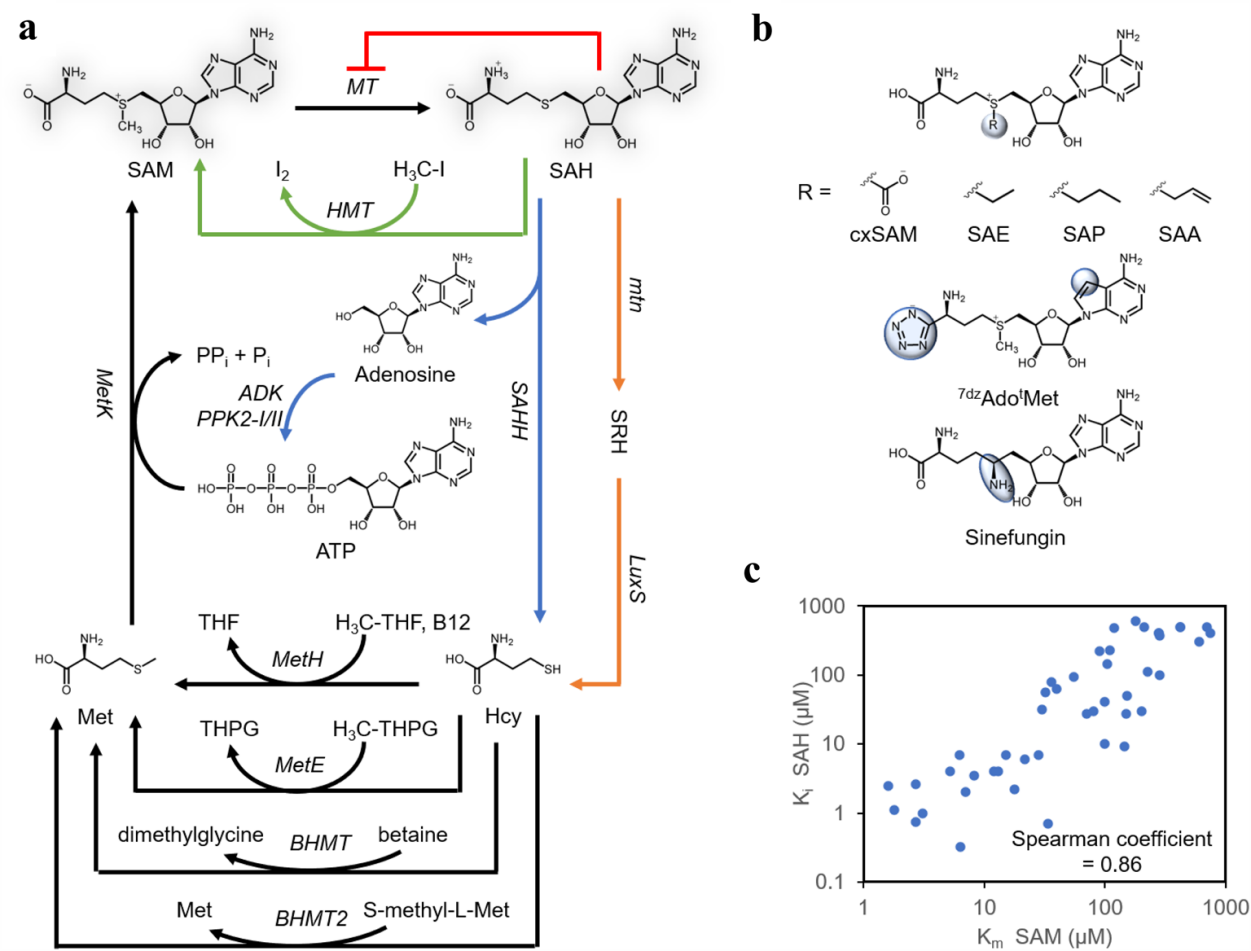
Co-factors for methyltransferase reaction. **a** Illustration of SAM regeneration reactions. **b** Illustration of SAM analogues. **c** *K*_i_ of SAH and the *K*_m_ of SAM for small molecule methyltransferases display a high correlation with spearman coefficient of 0.86 (refer to Table 3 for detailed information). The abbreviations are as follows. SAM, *S*-adenosylmethionine; SAH, *S*-adenosylhomocysteine; Met, l-methionine; Hcy, hmocysteine; SRH, *S*-(5-deoxy-d-ribos-5-yl)-l-homocysteine; THF, Tetrahydrofolate; THPG, tetrahydropteroyltri-l-glutamate; H3C-THF, 5-methyltetrahydrofolate; H3C-THPG, 5-methyltetrahydropteroyltri-l-glutamate; SAE,*S*-adenosyl-l-ethionine; SAA, *S*-allyl-l-homocysteine; SAP, *S*-propyl-l-homocysteine; l-^t^Met, tetrazole-l-methionine; ^7dz^ATP, 7-deaza-ATP; Pi, phosphate; PPi, pyrophosphate; MT, methyltransferase; HMT, halide methyltransferase; SAHH, SAH hydroxylase; Mtn, SAH nucleosidase; LuxS, *S*-ribosylhomocysteine lyase; metH, cobalamin (or viatamin B12)-dependent methionine synthase; metE, cobalamin (or viatamin B12)-independent methionine synthase; BHMT, Betaine–homocysteine S-methyltransferase 1; BHMT2, *S*-methylmethionine–homocysteine S-methyltransferase; ADK, adenosine kinase; PPK2-I/II, polyphosphate kinase; metK, *S*-adenosylmethionine synthase

In addition to improving methyltransferase activities, SAM recycling is another crucial aspect for biotechnological applications of methyltransferases; it removes SAH inhibition, regenerates otherwise unstable SAM and reduces costs (Mordhorst and Andexer 2020; Popadić et al. 2021). The native SAM recycling pathway involves multiple enzymatic reactions (Fig. 6a). One of the reactions involves complex co-factors, namely, methyl-tetrahydrofolate and vitamin B12 (cobalamin), rendering recycling SAM difficult extracellularly. As a result, in vivo SAM regeneration may be more viable or economical for SAM-dependent natural product biosynthesis (Bennett et al. 2017). In this section, recent advances in SAM regeneration, both in vitro and in vivo, are discussed. Finally, we will highlight the use of synthetic SAM analogues to transfer alternative chemical groups to diversifying natural products.

#### Increase SAM concentration

To develop a SAM recycling system in vitro, Popadic et al. systematically optimized a bicyclic regeneration system (Fig. 6a): one cycle employs adenosine kinase (ADK) and polyphosphate kinases (PPK2-I/II) to regenerate ATP from adenosine and the other cycle employs betaine-l-homocysteine S-methyltransferase (BHMT) to regenerate l-methionine (Met) from l-homocysteine (Hcy) and betaine (Popadić et al. 2021). The in vitro system requires seven enzymatic steps, which are challenging to be efficiently implemented (Mordhorst and Andexer 2020). Recently, Liao and Seebeck (2019) employed a reversible halide methyltransferase (HMT) from *Chloracidobacterium thermophilum* to produce SAM from SAH and methyl halide (Fig. 6a), demonstrating the first possible method to directly synthesize SAM from SAH in vitro. The co-factor recycling system has been successfully applied to multiple methyltransferases reactions in vitro, allowing > 90% conversion with catalytic amount of SAH. However, a high concentration of methyl halide is required, which poses as a safety hazard. Less hazardous methyl donor needs to be explored before implementing the SAM/SAH recycling system in a larger scale.

Instead of in vitro, in vivo SAM regeneration is often used. In the exponentially growing *E. coli* strain MG1655, SAM concentration was determined to be 0.4 mM (Parveen and Cornell 2011). To increase the intracellular SAM concentration in a heterologous host, l-methionine feeding with overexpression of *S*-adenosylmethionine synthetase (metK from *E. coli* or SAM2 from *S. cerevisiae*) has been applied (Hu et al. 2012; Han et al. 2016; Heo et al. 2017; Xu et al. 2019; Liu et al. 2019). To save the cost of production, Liu et al. (2019) engineered an industrial yeast strain to covert d-methionine to l-methionine by disrupting d-amino acid-N-acetyltransferase (HPA3), and overexpressing d-amino acid oxidase (DAAO) from *Trigonopsis variabilis* and l-phenylalanine dehydrogenase (l-PheDH) from *Rhodococcus jostii*. Thus, cheaper racemic dl-methionine can be supplemented in media and converted to SAM by overexpressed SAM2 enzyme. With genetic modifications to reduce SAM degradation, 10.3 g/L SAM was successfully produced by the engineered yeast strain in 10 L bioreactor with 16 g/L dl-methionine feeding.

SAM metabolism is highly regulated and subjected to feedback inhibition in *E. coli* (Cress et al. 2017). When SAM accumulates, it binds to the repressor *metJ* and transcriptionally represses genes that are responsible for SAM biosynthesis (Smith and Greene 1984). To improve the O-methylated anthocyanin production in *E. coli*, Cress et al. (2017) silenced *metJ* via CRISPRi-mediated deregulation. The strategy effectively increased O-methylated anthocyanin production by twofold, indicating SAM availability limits O-methyltransferase activity in *E. coli* (Cress et al. 2017). Similarly, deleting *metJ* together with overexpressing methionine biosynthetic pathway genes increased SAM concentration and improved vanillin production in *E. coli* by 33% (Kunjapur et al. 2016).

By applying the feedback regulation of SAM and metJ-metO system, Umeyama et al. (2013) constructed a genetic circuit in *S. cerevisiae* to report intracellular SAM concentration (Fig. 5c). The authors fused *metJ* with transcriptional activator domain (AD) B42 and incorporated a reporter gene downstream of methionine operator *metO*. In the presence of SAM, the SAM-metJ-B42 complex binds to *metO*, and B42 will activate the expression of downstream reporter gene. The authors have demonstrated the circuit was able to detect as low as 5 µM SAM. With this gene circuit as a high-throughput screen for SAM production, the study has identified that overexpressing GAL11 could improve SAM concentration by 3.3-fold. The same screening method was applied by Dong et al. (2021). The authors have established a MAGIC (multi-functional genome-wide CRISPR) method to simultaneously activate and interfere transcription, and delete genes. By multiple rounds of transforming guide RNA libraries (size > 10^6^) into the SAM sensing yeast strain, the authors identified novel targets, namely, the upregulation of SNZ3, RFC4 and RPS18B enhanced SAM accumulation by 2.2- and 1.6-fold in laboratory and industrial yeast strains, respectively.

#### Reduce SAH inhibition

While increasing SAM availability improves methyltransferase activity, alleviating the by-product SAH inhibition is another effective strategy (Dorgan et al. 2006). SAH has been reported to be a potent inhibitor of many methyltransferases; SAH binds to some methyltransferases stronger than SAM, and the *K*_i_ value of SAH reported could be as low as sub-micromolar range (Table 3) (Petronikolou and Nair 2015). Accumulation of SAH in vivo is toxic (Christopher et al. 2002; James et al. 2002). In some microorganisms, sophisticated regulatory system has been evolved to sense and prevent accumulation of SAH intracellularly. For example, an RNA riboswitch has been identified in *Pseudomonas syringae* upstream of SAH degradation pathway enzymes (Wang et al. 2008). The riboswitch forms a 3D structure that conceals or exposes the translation initiation site in the absence or presence of SAH, respectively, thus effectively maintaining intracellular concentration of SAH below micromolar range. Similar regulatory elements have been identified in proteobacteria, actinobacteria and others. Such riboswitches have been modified and utilized as biosensors to monitor methyltransferase activity in vivo (Su et al. 2016).

**Table 3:** Summary of *K*_m,SAM_ and *K*_i,SAH_ of all the small molecule methyltransferase in BRENDA

EC number	Name	Organism	*K*_m_ (µM SAM)	*K*_i_ (µM SAH)	Refs.
2.1.1.6	Catechol O-methyltransferase	*Homo sapiens*	3.1	1	Rivett and Roth (1982)
2.1.1.6	Catechol O-methyltransferase	*Candida tropicalis*	6.2	6.9	Veser (1987)
2.1.1.15	Fatty-acid O-methyltransferase	*Mycobacterium marinum*	33.6^a^	0.7^a^	Petronikolou and Nair (2015)
2.1.1.20	Glycine N-methyltransferase	*Oryctolagus cuniculus*	200	30	Kloor et al. (2004)
2.1.1.79	Cyclopropane-fatty-acyl-phospholipid synthase	*Escherichia coli*	90	220	Taylor and Cronan (1979)
2.1.1.95	Tocopherol C-methyltransferase	*Arabidopsis thaliana*	5.2	4uM^b^	Koch et al. (2003)
2.1.1.106	Tryptophan 2-C-methyltransferase	*Streptomyces laurentii*	120	480	Frenzel et al. (1990)
2.1.1.142	Cycloartenol 24-C-methyltransferase	*Glycine max*	32	56	Nes et al. (2003)
2.1.1.156	Glycine/sarcosine N-methyltransferase	*Halorhodospira halochloris*	280 with sarcosine	400^c^	Nyyssölä et al. (2001)
			420 with glycin	500^c^	
2.1.1.156	Glycine/sarcosine N-methyltransferase	*Aphanothece halophytica*	700 with glycin	500	Waditee et al. (2003)
			600 with sarcosine	300	
		*Ectothiorhodospira halochloris*	420 with glycin	500	
			280 with sarcosine	400	
		Rat	36 with glycin	80	
2.1.1.157	Sarcosine/dimethylglycine N-methyltransferase	*Aphanothece halophytica*	180	600	Waditee et al. (2003)
		*Ectothiorhodospira halochloris*	210 with sarcosine	500	
2.1.1.165	Methyl halide transferase	*Brassica oleracea*	30	32	Attieh et al. (1995)
2.1.1.7	Nicotinate N-methyltransferase	*Glycine max*	55	95	Upmeier et al. (1988)
2.1.1.8	Histamine N-methyltransferase	Human	1.8	1.1	Francis et al. (1980)
2.1.1.9	Thiol S-methyltransferase	Rat	105	144	Borchardt and Cheng (1978)
2.1.1.25	Phenol O-methyltransferase	*Phanerochaete chrysosporium*	99	41	Coulter et al. (1993)
2.1.1.46	Isoflavone 4′-O-methyltransferase	*Cicer arietinum*	80	30	Wengenmayer et al. (1974)
2.1.1.50	Loganate O-methyltransferase	*Catharanthus roseus*	742.1	400	Murata et al. (2008)
2.1.1.53	Putrescine N-methyltransferase	*Datura stramonium* L.	100	10	Walton et al. (1994)
2.1.1.53	Putrescine N-methyltransferase	*Hyoscyamus albus*	227	110	Hibi et al. (1992)
2.1.1.67	Thiopurine S-methyltransferase	*Homo sapiens*	2.7	0.75	Woodson and Weinshilboum (1983)
2.1.1.68	Caffeate O-methyltransferase	*Beta vulgaris* L.	13	4	Poulton and Butt (1975)
2.1.1.68	Caffeate O-methyltransferase	*Glycine max*	15	6.9	Poulton et al. (1976)
2.1.1.68	Caffeate O-methyltransferase	*Medicago sativa *L.	7	2	Edwards and Dixon (1991)
2.1.1.68	Caffeate O-methyltransferase	*Medicago sativa *L.	12	4	Edwards and Dixon (1991)
2.1.1.91	Isobutyraldoxime O-methyltransferase	*Pseudomonas *sp.* N.C.I.B. 11652*	150	27	Harper and Kennedy (1985)
2.1.1.94	Tabersonine 16-O-methyltransferase	*Catharanthus roseus*	21.7	6	Levac et al. (2008)
2.1.1.102	Demethylmacrocin O-methyltransferase	*Streptomyces fradiae*	110	226	Kreuzman et al. (1988)
2.1.1.104	Caffeoyl-CoA O-methyltransferase	*Petroselinum crispum*	8.2	3.5	Pakusch and Matern (1991)
2.1.1.103	Phosphoethanolamine N-methyltransferase	*Caenorhabditis elegans*	145	9.1	Brendza et al. (2007)
2.1.1.103	Phosphoethanolamine N-methyltransferase	*Plasmodium falciparum*	153	50^c^	Pessi et al. (2004)
2.1.1.107	Uroporphyrinogen-III C-methyltransferase	*Pseudomonas denitrificans*	6.3	0.32	Blanche et al. (1989)
2.1..1.136	Chlorophenol O-methyltransferase	*Trichoderma longibrachiatum*	284	368.9	Coque et al. (2003)
2.1.1.140	(*S*)-Coclaurine-N-methyltransferase	*Tinospora cordifolia*	40	62	Loeffler et al. (1995)
2.1.1.147	Corydaline synthase	*Corydalis cava*	2.7	2.6	Rueffer et al. (1994)
2.1.1.153	Vitexin 2″-O-rhamnoside 7-O-methyltransferase	*Avena sativa *L.	1.6	2.5	Knogge and Weissenböck (1984)
2.1.1.154	Isoliquiritigenin 2′-O-methyltransferase	*Medicago sativa *L.	17.7	2.2	(Medicago sativa L.) Maxwell et al. (1992)
2.1.1.273	Benzoate O-methyltransferase	*Antirrhinum majus*	28	7	Murfitt et al. (2000)
2.1.1.338	Desmethylxanthohumol 6′-O-methyltransferase	*Humulus lupulus*	286	98	Nagel et al. (2008)
2.1.1.343	8-Amino-8-demethylriboflavin N,N-dimethyltransferase	*Streptomyces davaonensis*	70	27	Tongsook et al. (2016)

^a^Kd

^b^Concentration when inhibition observed

^c^IC_50_ or 50% inhibition

To our knowledge, directed enzyme engineering methods to mutate methyltransferases and alleviate SAH inhibition have not been investigated. In general, SAH shares the same binding pocket as SAM, and hence, SAH is often used to co-crystalize with methyltransferases to probe the co-factor binding site (Liscombe et al. 2012). Mutating residues interacting with SAH will invariably affect the binding affinity of SAM. This is evident from the linear correlation between the *K*_m_ of SAM and the *K*_i_ values of SAH, with Spearman coefficient of 0.86 (Fig. 6c and Table 3). A more common strategy is to co-express enzymes in the methionine cycle, SAH nucleosidase (mtn) or SAH hydrolase (SAHH), to remove SAH. Kunjapur and co-authors applied the strategy to increase vanillin production in *E. coli* (Kunjapur et al. 2016). Interestingly, vanillin titer was only improved when mtn and LuxS from *E. coli* were co-expressed, indicating that SAH removal enhanced methyltransferase activity. Surprisingly, when SAHH from *S. cerevisiae* was co-expressed, vanillin titer was reduced. The unexpected detrimental effect of overexpressing SAHH on vanillin titer was possibly because of the poor expression of SAHH from eukaryotic origin. Screening of SAHH activities from bacterial origin could be tested. Coupling SAH degradation to SAM generation will be ideal to increase co-factor supply and reduce feedback inhibition to methyltransferases. Optimizing enzymatic activities along the methionine cycle may be beneficial to maximize SAM and minimize SAH concentration.

#### Diversification with SAM analogues

The versatility of methyltransferases has led to creative applications, such as transferring more complex moiety to biomolecules, and SAM analogues have been developed in recent years (Dalhoff et al. 2006; Singh et al. 2014). Singh et al. (2014) screened five methionine adenosyltransferases (MATs) and identified human MAT II could synthetize 29 non-native SAM analogues from _L_-methionine analogues and ATP. By coupling MAT II with rebeccamycin methyltransferase (RebM), differentially alkylated indolocarbazoles were produced in appreciable yields (≥ 40%). Moreover, extending the application of halide methyltransferase (HMT), Tang et al. (2021) mutated an HMT from *Arabidopsis thalia* to transfer ethyl-, propyl- and allyl-moieties to SAH and produce corresponding SAM analogues with high efficiency (Fig. 6b). Recently, a naturally occurring SAM analogue biosynthesis pathway, the carboxy-*S*-adenosyl-methionine (cxSAM) pathway, was discovered; CmoA catalyses the transfer of carboxylic group from prephenate to the methyl group in SAM, producing cxSAM (Kim et al. 2013; Herbert et al. 2020) (Fig. 6b). This opens up the possibility to generate diverse carboxymethylated products both in vitro and in vivo. One caveat is that carboxymethylation is an extremely rare reaction in nature and wild type methyltransferases may not readily accept cxSAM as the co-factor. CmoA was discovered to work in tandem with a tRNA carboxymethyltransferase (CmoB) which exhibits 500-fold higher affinity towards cxSAM over SAM (Herbert et al. 2020). By carefully examining the co-factor binding residues from the X-ray crystal structure of CmoB, Herbert and colleagues have identified key residues to mutate in a catechol-O-methyltransferase (COMT) from *Rattus norvegicus* and a coclaurine-N-methyltransferase (CNMT) from *Coptis japonica* so as to engineer these methyltransferases to be more selective with cxSAM than SAM (Herbert et al. 2020). By coupling these engineered methyltransferases with CmoA, carboxymethylated products, namely, carboxymethylated catechol and carboxymethylated tetrahydroisoquinoline, have been produced by COMT or CNMT, respectively.

In addition, a few SAM analogues have improved stability as compared to SAM, which is susceptible to depurination, intramolecular cyclization and sulfonium epimerization. To stabilize SAM, Huber et al. (2016) have synthesized an analogue of SAM, ^7dz^Ado^t^Met, from ^7dz^ATP and l-^t^Met (Fig. 6b). The analogue exhibits exceptionally high stability at pH8. Interestingly, permissive carminomycin 4-OMT (DnrK) can utilize the modified SAM analogue with similar efficiency as SAM, thus potentially ^7dz^Ado^t^Met can be an advantageous substitute in SAM-dependent enzymatic reaction. Although SAM analogues hold a great promise to diversify natural products, their synthesis and regeneration still pose a challenge for scaled-up applications.

## Conclusions

With the advancements in genomic and chemical screening methods, novel SAM-dependent methyltransferases have been discovered. Many of methyltransferases are ubiquitous in various natural product biosynthetic pathways, and sometimes a network of methyltransferases participates concertedly in diversifying the natural product. Structural elucidation of these methyltransferases along the same pathway will provide valuable insights on their specificity and advance our understanding of how these methyltransferases are permissive to accept structurally similar compounds and at the same time specific to the site of methylation. The knowledge will allow us to better predict enzyme function and alter enzyme specificity (Morris et al. 2020). Notably, many SAM-dependent methyltransferases reviewed here are catalytically promiscuous. These are excellent initial templates to methylate non-native molecules and potentially allow retrosynthetic design of artificial pathway. Side activities are often inefficient and require substantial engineering efforts. High-throughput assays coupled with computer-aided enzyme design will significantly speed up the optimization process. Integrated assays can be applied to narrow down the library of beneficial mutants: the growth-coupled methyltransferase assay can be applied first to screen out non-active methyltransferase mutants. Subsequently, SAH biosensor can be applied to the active mutants to identify higher methyltransferase activities. Application of the biosensor remains to be demonstrated for methyltransferase engineering. Despite the exciting development in methyltransferase discovery and engineering, to apply methyltransferases as industrial enzymes, co-factor regeneration is still a challenge. Biosynthesis of SAM from SAH via methyl halide methyltransferase is a breakthrough in SAM co-factor regeneration in vitro. However, its application is limited by the safety concern of methyl iodide. Thus, more innovation is required to identify novel SAM recycling enzymes.

Taken together, the field of SAM-dependent methyltransferases has advanced significantly with the discovery of novel methyltransferases and innovative solutions to improve methyltransferase activity and diversify methyltransferase reaction. These open up the possibility of biosynthesizing individual complex natural product, especially some natural products that cannot be obtained in pure form and contaminants from isomers are toxic (Li et al. 2018). Moreover, the expanded biocatalytic property of methyltransferases to catalyse non-native substrate/co-factors will widen the scope of chemical diversity which can be explored for food, flavour and fragrance, energy, and pharmaceutical industries.

## Data Availability

Not applicable.
